# Case report: Multiple biventricular aneurysms in arrhythmogenic cardiomyopathy

**DOI:** 10.3389/fcvm.2022.1034703

**Published:** 2023-01-24

**Authors:** Jiadong Lin, Zhijuang Lu, Mingqin Lin, Ying Wan, Jianfeng Li, Xinsheng Huang

**Affiliations:** ^1^Ultrasound Department, Dongguan Hospital of Guangzhou University of Chinese Medicine, Dongguan, China; ^2^Cardiovascular Department, Jinshazhou Hospital of Guangzhou University of Chinese Medicine, Guangzhou, China

**Keywords:** arrhythmogenic cardiomyopathy, echocardiography, desmoplakin, high-frequency ultrasound, sustained ventricular tachycardia, syncope

## Abstract

Arrhythmogenic cardiomyopathy (ACM) is a genetic disease characterized by fibro-fatty myocardial replacement and is clinically associated with malignant ventricular arrhythmias and sudden cardiac death. It presents a major diagnostic and therapeutic challenge due to its complex clinical presentation and multiparametric diagnostic scoring system that includes structural, histological, and electrocardiographic data. A 57-year-old man with a history of palpitation and premature ventricular contractions (PVC) experienced syncope and sustained ventricular tachycardia at a rate of 213 bpm, which was successfully rescued by synchronized cardioversion. Multiple ventricular aneurysms were found in the right ventricular free wall and the left ventricular apical regions, as well as mild biventricular systolic dysfunction, according to echocardiography and high-frequency ultrasound. The genetic analysis revealed the following desmoplakin genes, chr6-7585274-7585275, NM_004415, exon24, and c.7780delT (p.S2594Pfs^*^9), a heterozygous and likely pathogenic mutation, as the mutation sites in the patient and his 24-year-old daughter. During the 21-month follow-up, the patient did not experience syncope or pre-syncope symptoms while on *β*-blocker (bisoprolol) therapy. Among the multimodality imaging techniques of the ACM, late gadolinium enhancement on cardiac magnetic resonance (CMR) is accepted as a more objective indicator of myocardial fibrosis. Left ventricular systolic dysfunction, fibrosis on CMR, and frequent PVC are the primary and most sensitive clinical signs of desmoplakin cardiomyopathy. However, echocardiography continues to be the most commonly used imaging modality for assessing focal ventricular movement and structural abnormalities. The pathological characteristics of arrhythmogenic cardiomyopathy of the right ventricular anterior free wall and apical regions near the transducer can be better shown using high-frequency linear ultrasound with a higher resolution.

## Introduction

An international task force proposed diagnostic criteria for arrhythmogenic right ventricular cardiomyopathy (ARVC) in 1994, based on identifying structural abnormalities, fatty or fibro-fatty replacement of the right ventricular myocardium, electrocardiographic changes, arrhythmias of right ventricular origin, and familial disease (1). In 2010, these criteria were revised to improve sensitivity for early diagnosis (2). A definite, borderline, or possible diagnosis of ARVC was established based on the fulfillment of major and/or minor criteria. Padua's diagnostic criteria for arrhythmogenic cardiomyopathy included dominant-right, biventricular, and dominant-left phenotypes (ACM) (3). The clinical profiles of asymptomatic family members with concealed structural abnormalities and no arrhythmias in symptomatic patients experiencing arrhythmic cardiac arrest or requiring a heart transplant because of refractory heart failure are only a few examples of how the phenotypic expression of ACM can differ (4). Diagnosis of ACM remains difficult due to the lack of a single gold standard diagnostic tool, poor specificity of ECG findings, multiple possible causes of right ventricular arrhythmias, difficulty evaluating the right ventricle using imaging, and occasional inconclusiveness of the pathogenicity of genetic variants detected (4). In this case report, we describe the case of a patient with ACM who had multiple ventricular aneurysms in both ventricles and mild biventricular systolic dysfunction. Although only 17% of probands had focal right ventricular sacculations (5), the sacculation may be a relatively specific echocardiographic phenotype of ACM.

## Timeline

**Table:** 

**Date**	**Progress**
2013	A history of palpitation with normal physical examination findings.
2019	Electrocardiographic frequent PVC.
November 2020	Sustained ventricular tachycardia with 213 bpm was immediately terminated by synchronized cardioversion. The coronary angiogram was normal. Holter monitoring showed 4,082 (4.4%) frequent different morphological PVC.
November 2020	Echocardiography showed multiple biventricular aneurysms with mild systolic dysfunction.
December 2020	The mutation site was the desmoplakin gene, likely a pathogenic mutation.
February 2021	Holter monitoring showed 4,065 (4.4%) frequent PVC.
May 2022	Echocardiographic findings were similar to November 2020. The patient did not experience syncope during the 21-month follow-up.

## Case description

**Figure 1:**
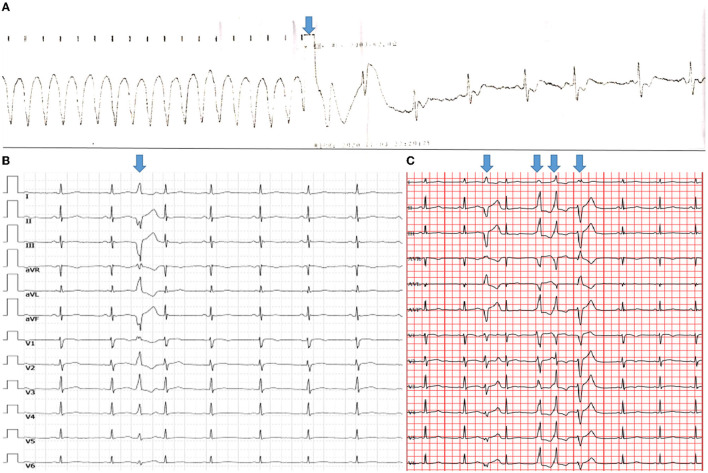
Ventricular arrhythmias. The height and weight of the patient were 170 cm and 55 kg, respectively. **(A)** Electrocardiography revealed that synchronized cardioversion restored ventricular tachycardia to sinus arrhythmia (arrow). **(B)** A 12-lead standard electrocardiogram revealed relatively low QRS-complex voltage at all leads, as well as a single PVC with RBBB morphology and a superior axis, indicating that the PVC originated in the left ventricle. **(C)** Holter demonstrated various morphological singles and triplets of PVC (arrows) that may have originated from different sites.

In 2013, a 57-year-old man had palpitation with normal physical examination findings. He was diagnosed with frequent premature ventricular contractions (PVCs) in 2019 despite not taking the antiarrhythmic medication regularly. He had palpitations, dyspnea, and sweating, followed by syncope during emotional disturbance, and regained consciousness ~10 minutes later, in November 2020. He experienced another loss of consciousness and hemodynamic instability in the emergency department of an outside hospital, with a blood pressure of 84/50 mmHg. The 213 bpm electrocardiographically sustained ventricular tachycardia was quickly terminated by synchronized cardioversion (Figure 1A). Because of his normal coronary angiogram and echocardiographically normal systolic function without structural heart disease, he was diagnosed with idiopathic ventricular arrhythmias.

The patient was referred to our institute 20 days later. The 12-lead standard electrocardiogram revealed a single PVC with right bundle branch block (RBBB) morphology and a superior axis, indicating that the PVC originated in the left ventricle (Figure 1B). In November 2020, Holter monitoring revealed 4,082 (4.4%) frequent different morphological PVCs, including 165 couplets and 2 triplets. Different morphological PVCs have been identified, which may have originated in different regions (Figure 1C). Based on observational data and expert consensus, the patient was referred for appropriate radiofrequency ablation for PVC and/or implantable cardioverter defibrillator therapy for fast ventricular tachycardia. Despite informed consent, the patient refused radiofrequency ablation and/or an implantable cardioverter defibrillator. β-Blocker (bisoprolol) therapy was administered to the patient during the follow-up to alleviate symptoms.

Holter monitoring revealed 1,226 (1.5%) frequent PVCs in December 2020, including 30 couplets, and 4,065 (4.4%) frequent PVCs in February 2021, including 131 couplets and 1 triplet, with no non-sustained ventricular tachycardia over three consecutive PVCs.

Following the collection of clinical characteristics, electrocardiograms, echocardiograms, and laboratory examinations, a genetic analysis was performed. The peripheral venous blood samples of the patient and his 24-year-old daughter were sent to the medical laboratory of Beijing Mygenostics Co., Ltd. for relevant gene capture, gene enrichment, and Sanger sequencing. The desmoplakin genes, chr6-7585274-7585275, NM_004415, exon24, c.7780delT (p.S2594Pfs^*^9), a heterozygous and likely pathogenic mutation, were the sites of their mutation.

**Figure 2:**
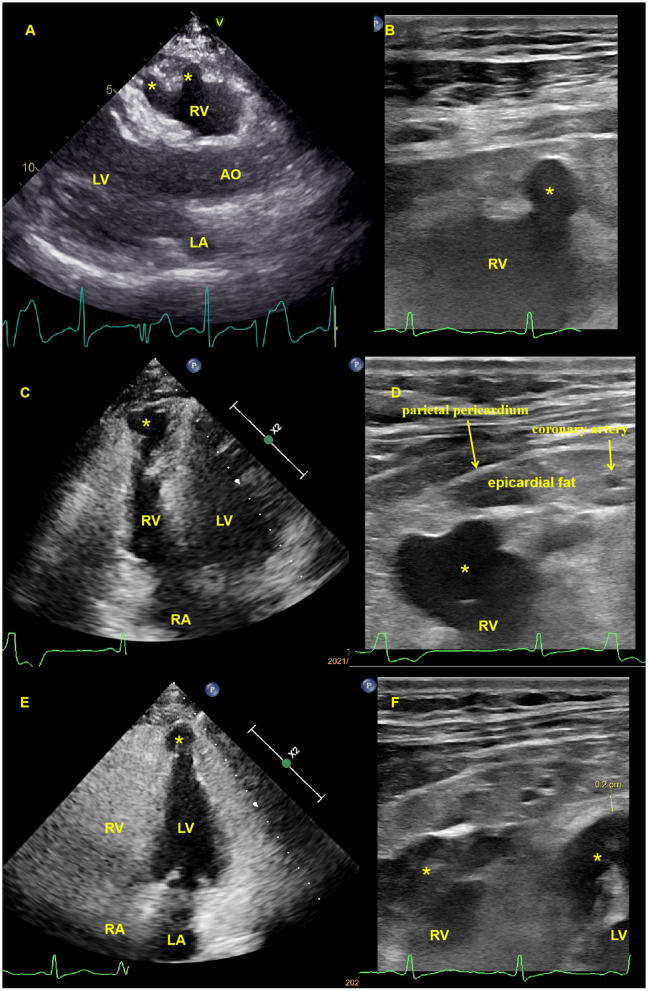
Transthoracic echocardiography and high-frequency linear ultrasound showed multiple biventricular aneurysms. **(A)**. Transthoracic echocardiography revealed an anterior right ventricular free wall aneurysm (asterisk) in the parasternal long axis view (see Supplementary Video 1), which had previously been identified using high-frequency linear ultrasound **(B, C)**. Transthoracic echocardiography revealed a right ventricular apical free wall aneurysm (asterisk) in the apical four-chamber view that was demonstrated by high-frequency linear ultrasound **(D)**, (see Supplementary Video 2), with a yellow arrow pointing to the apical epicardial coronary artery and a red arrow pointing to the parietal pericardium **(E)**. Transthoracic echocardiography revealed a left ventricular apical aneurysm (asterisk) in the apical four-chamber view, and high-frequency linear ultrasound revealed both ventricular apical aneurysms **(F)**, (asterisk) with an apical left ventricular thickness of 2 mm. High-frequency ultrasound can also reveal ventricular aneurysmal wall thickness, apical coronary artery, epicardial fat, and parietal pericardial thickness **(B, D, F)**. AO, aorta; LA, left atrium; LV, left ventricle; RA, right atrium; RV, right ventricle.

The first transthoracic echocardiographic examination of our institute was performed in November 2020, and subsequent echocardiography was performed in January 2021, July 2021, and May 2022, respectively. With mild biventricular systolic dysfunction (left ventricular ejection fraction of 47% and right ventricular ejection fraction of 40%), echocardiography revealed similar results with multiple ventricular aneurysms in the right ventricular free wall and left ventricular apical regions. The high-frequency ultrasound images were obtained using a commercial Philips EPIQ 7C ultrasound machine (Philips Medical Systems, Andover, MA, USA) equipped with a 3–12 MHz high-frequency linear transducer probe with a vascular carotid preset. The aneurysm measured by two-dimensional echocardiography was 13 (width) × 10 (depth) mm in the right ventricular apical free wall, 7 × 9 and 11 × 16 mm in the right ventricular anterior free wall, and 15 × 19 mm in the left ventricular apical region. High-frequency linear ultrasound with a higher resolution could better show and measure ventricular aneurysms near transducer regions (Figure 2). The patient was diagnosed with arrhythmogenic cardiomyopathy (ACM) after having echocardiographic multiple ventricular aneurysms and mild biventricular systolic dysfunction, after having sustained ventricular tachycardia, when diagnosed with >500 ventricular extrasystoles per 24 h (Holter), and upon the identification of a pathogenic mutation categorized as associated or probably associated with ACM (2, 3) or was diagnosed with arrhythmogenic cardiomyopathy (ACM) with a phenotype of multiple biventricular aneurysms. The echocardiography, 12-lead standard electrocardiogram, and Holter analysis of his daughter were all normal. During the 21-month follow-up, the patient did not present with syncope.

## Discussion

Arrhythmogenic cardiomyopathy is believed to be a heredo-familial cardiac disease characterized by fibro-fatty myocardial replacement and an increased risk of sudden cardiac death. It is a heritable, progressive cardiac disease with a wide range of phenotypes. Although ARVC is the most common phenotype, several other ACM phenotypes have been recognized and described (1–3). Desmoplakin cardiomyopathy is increasingly being recognized in distinct arrhythmogenic cardiomyopathies with prominent involvement of the left ventricle, as opposed to the classical forms of ARVC (6). We present a patient with the phenotype of multiple biventricular aneurysms; it seems that the morphological abnormality of the right ventricle is more visible than that of the left ventricle. On the 12-lead standard electrocardiogram and Holter record, there were several morphological PVCs. The different morphological PVCs may have originated from different regions.

Although echocardiography is the most commonly used imaging technique, cardiac magnetic resonance (CMR) is the preferred initial test for patients with suspected ARVC due to its accuracy, availability, safety, and low cost (7). Among the multimodality imaging of the ACM, late gadolinium enhancement on CMR is accepted as a more objective indicator of myocardial fibrosis. Left ventricular systolic dysfunction, fibrosis on CMR, and frequent PVCs are the primary and most sensitive clinical signs of desmoplakin cardiomyopathy (6). CMR may increase the risk of ACM misdiagnosis due to the difficult and operator-dependent interpretation of RV wall motion abnormalities, which are the major diagnostic criteria (8).

When evaluating ventricular wall motion, echocardiography has a better temporal resolution than CMR. The high-frequency linear ultrasound with superior spatial and temporal resolution revealed a focal ventricular aneurysm and abnormal ventricular motion in the right ventricular anterior free wall and apical regions near the transducer. However, high-frequency ultrasound with higher attenuation has a limited penetration depth; the optimal exploration distance is <3–4 cm from the transducer to the region of interest.

To the best of our knowledge, this is the first case report that uses high-frequency ultrasound to assess a focal ventricular aneurysm associated with arrhythmogenic cardiomyopathy. It can better demonstrate the pathological characteristics of arrhythmogenic cardiomyopathy near the probe.

## Data availability statement

The original contributions presented in the study are included in the article/Supplementary material, further inquiries can be directed to the corresponding authors.

## Ethics statement

Ethical review and approval was not required for the study on human participants in accordance with the local legislation and institutional requirements. The patients/participants provided their written informed consent to participate in this study. Written informed consent was obtained from the individual for the publication of any potentially identifiable images or data included in this article.

## Author contributions

The patient data were managed, analyzed, and interpreted by JLin and XH. JLin and XH made significant contributions to the composition of the manuscript. The manuscript was written by ZL and ML. The treatment and follow-up of the patient were handled by YW and JLi. The final written manuscript was reviewed and approved by all authors.
